# Renal Mucormycosis: A Rare and Potentially Lethal Complication of Kidney Transplantation

**DOI:** 10.1155/2013/915423

**Published:** 2013-10-22

**Authors:** SreyRam Kuy, Chun He, David C. Cronin

**Affiliations:** ^1^Division of Vascular Surgery, Department of Surgery, Medical College of Wisconsin, 9200 W Wisconsin Avenue, Milwaukee, WI 53226, USA; ^2^Department of Pathology, Medical College of Wisconsin, Milwaukee, WI, USA; ^3^Division of Transplantation, Department of Surgery, Medical College of Wisconsin, Milwaukee, WI, USA

## Abstract

Renal mucormycosis is a rare and potentially lethal complication of kidney transplantation. We describe two cases of renal mucormycosis following deceased donor kidney transplantation. This is the second report of renal mucormycosis following kidney transplantation in the United States, and the first case of renal mucormycosis infection presumed to be of recipient origin. Case A had an early presentation of mucormycosis isolated to the kidney allograft. He had an unexpected rise in serum creatinine and leukocytosis necessitating allograft biopsy which showed mucormycosis. He underwent transplant nephrectomy on posttransplant day 11, was treated with amphotericin B, and discharged home on posttransplant day 22. Case B had a late presentation of renal mucormycosis, preceded by a cutaneous manifestation. One year after kidney transplantation he had a nonhealing knee ulcer which on biopsy showed cutaneous mucormycosis. Treatment included aggressive debridement and amphotericin B. Allograft biopsy showed mucormycosis, necessitating transplant nephrectomy. He was discharged to a rehabilitation facility and died from noninfectious causes. Review of the published literature of renal mucormycosis cases following kidney transplantation reveals a mortality rate of more than 50%. The key to successful outcome is early recognition, prompt institution of surgical debridement of all infected tissue, and appropriate antifungal therapy.

## 1. Introduction

Mucormycosis, previously known as zygomycosis, refers to rare infections caused by fungi of the order of *Mucorales*, which are characterized by the production of aseptate hyphae and asexual spores [1]. The most common clinical isolate of *Mucorales* is *Rhizopus oryzae* (44%), followed by *Rhizopus microsporus* (22%), *Mucor circinelloides* (9.5%), *Mycocladus corymbifer* (5.3%), *Rhizomucor pusillus* (3.7%), *Cunninghamella bertholletiae* (3.2%), *Mucor indicus* (2.6%), *Cunninghamella echinulata* (1%), and *Apophysomyces elegans* (0.5%) [2]. Mortality among patients with mucormycosis is significant. Mortality was as high as 84% in the 1950s and then decreased to approximately 40% in the 2000s, with the drop in mortality attributed to the widespread introduction of amphotericin B in the 1960s [3]. However, from 1940 to 2000, there has been a 70% increase in the number of cases of mucormycosis, with mucormycosis most frequently seen in diabetic patients, followed by neutropenic patients, transplant recipients, patients with hematological disease, patients on deferoxamine therapy, and IV drug users. The most common presentation of mucormycosis is rhinocerebral disease, followed by pulmonary, cutaneous, and disseminated diseases. Less commonly, mucormycosis can involve the GI tract and kidneys [4, 5].

In contrast to the presentation of infection among nontransplant recipients, the majority of infections in solid-organ transplant recipients present as pulmonary (37%), followed by rhinocerebral (16%) and cutaneous (16%) infections. The Transplant-Associated Infection Surveillance Network (Roden) performed prospective surveillance for invasive fungal infections by routine review of monthly logs from a registry of solid organ transplant patients (liver, kidney, lung, pancreas, heart, and small bowel) at 15 US transplant centers from 2001 to 2006 [6]. The most common invasive fungal infections following solid organ transplantation were invasive candidiasis (53%), invasive aspergillosis (19%), cryptococcosis (8%), non-*Aspergillus *molds (8%), endemic fungi (5%), and, least commonly, mucormycosis (2%). The majorities of these mucormycosis cases were pulmonary (56%), with the remainder being sinus (13%), cutaneous (13%), and disseminated (9%). The incidence of invasive fungal infections was the highest among the small bowel transplant recipients (11.6%) and the lowest among kidney transplant recipients (1.3%). 

The first reported case of mucormycosis following kidney transplantation was a lethal rhinocerebral form in 1970 [7]. Among kidney transplant recipients, mucormycosis comprises only 2–6% of invasive fungal infections [6, 8]. However, compared with all other fungal infections mucormycosis is associated with the longest duration of hospitalization and highest 2-year mortality among kidney transplant patients [8]. Renal mucormycosis following kidney transplantation has been reported only once before in the United States. Though rare, renal mucormycosis is associated with 50% mortality among kidney transplant patients. 

We describe the second report of renal mucormycosis following kidney transplantation in the United States, and the first case of renal mucormycosis infection presumed to be of recipient origin. The remainder of the world literature describes 16 cases of renal mucormycosis following kidney transplantation, nearly all late occurrences of this complication. Overall, mucormycosis is uncommon, mucormycosis of the kidney is even more uncommon, and mucormycosis of the renal allograft specifically is both uncommon and usually a late occurring infection.

## 2. Two Case Reports

### 2.1. Case A

Case A, a 36-year-old man whose initial cause of end stage renal disease was Hemolytic uremic syndrome. His history is significant for receiving a deceased donor kidney transplant in 1991. In 1992 he underwent bilateral native nephrectomy and in 2007 a transplant nephrectomy was performed for chronic allograft nephropathy. Significant comorbidities include infection with hepatitis C virus (grade 1, stage 0). 

He received zero mismatch, cross match negative, a standard criteria deceased donor kidney transplant after 21 hours of cold preservation. Induction immunosuppression included antithymocyte globulin (rabbit, 1.5 mg/kg) and glucocorticoids. The kidney was implanted into a left lower quadrant retroperitoneal pocket. Due to a short donor ureter an ureteroureterostomy with internal stent provided drainage. After reperfusion the transplanted kidney was pink with firm turgor. 

**Figure 1:**
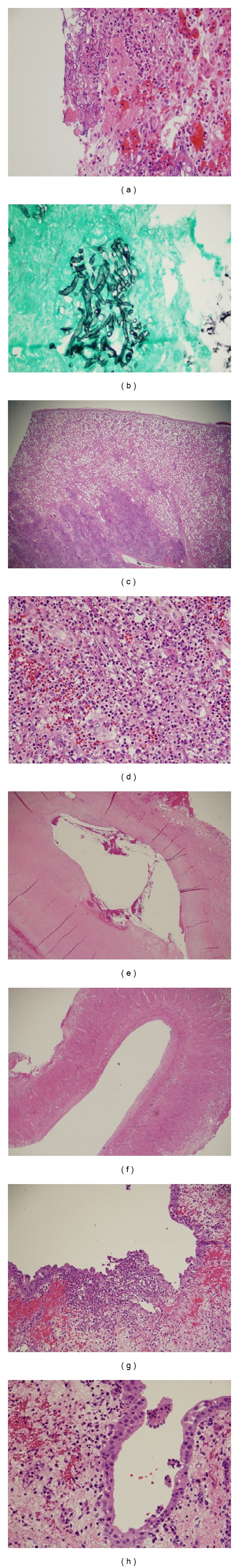
Case A renal allograft histology. Figures 1(a) and 1(b) show renal allograft biopsy indicating broad, pleomorphic, and thin walled fungal small fragments and hyphae with right angle branching and mucoid feature in presence of tissue necrosis consistent with mucormycosis in hematoxylin and eosin stain (a) and Gomori's methenamine silver stain (b). Explanted allograft kidney demonstrates diffuse and invasive mucormycosis predominantly involving the medulla noted by low magnification in (c) and high magnification in (d) on H&E stain; no mucormycetes invasion of renal artery (e) and vein (f); mucormycetes invasion in proximal portion of ureter indicative of donor ureter (g), and no mucormycetes invasion in distal portion of ureter indicative of recipient ureter (h).

On posttransplant (PT) day 1 there was scant urine output and the patient required hemodialysis. Urine output increased until postoperative day 4 when a decrease in urine output was associated with pain over the left lower quadrant and left groin. On PT 8, ultrasound was obtained due to a rising creatinine, which showed a migrated ureteral stent with hydronephrosis and he underwent cystoscopy with ureteral stent removal. Ultrasound on PT 9 showed persistent hydronephrosis and a nephrostomy tube was placed on PT 10. Nephrostogram on PT 11 showed the nephrostomy tube in proper position, but obstruction of the kidney. He had a persistent leukocytosis and fevers, but blood and urine cultures were negative for growth of aerobes and anaerobes. Due to concerns for rejection, he underwent allograft biopsy which showed mucormycosis and necrosis (Figure 1). He underwent an emergent total transplant nephrectomy including the ureter on PT 11 and was started on a course of amphotericin B. Histology of the explanted graft showed invasive mucormycosis of the transplanted kidney parenchyma and both donor and recipient ureter with an invasion pattern advancing from the luminal surface. There was no invasion of the graft renal vein or artery (Figure 1). This was suggestive of the recipient's urinary tract as the primary source for the mucormycosis. *Rhizomucor* was isolated from the specimen (speciation not identified). 

Cystoscopy done 4 days after nephrectomy showed normal appearing bladder with no evidence of necrosis. Due to the known predilection for pulmonary and rhinocerebral manifestations of mucormycosis, CT imaging of the lungs, sinuses, and brain were done which showed no evidence of mucormycosis. The patient was discharged home on PT 21.

Return to clinic on postnephrectomy day 25 revealed drainage from the incision. The patient underwent surgical debridement and wound exploration. Pathology showed no evidence of cutaneous mucormycosis in the wound. The organ procurement organization was promptly notified of this case of renal mucormycosis at the time of initial discovery. Neither the recipient of the partner kidney graft nor liver graft has demonstrated any evidence of mucormycosis. 

### 2.2. Case B

 Case B is a 54-year-old man with end-stage renal disease due to diabetes mellitus and hypertension. The patient underwent an expanded criteria deceased-donor, cross match negative kidney transplantation. Induction immunosuppression included antithymocyte globulin (rabbit, 1.5 mg/kg) and glucocorticoids. The initial postoperative course was complicated by delayed graft function, an upper extremity DVT, and a cardiac ischemic episode. One month after transplant the patient was seen for followup in clinic and noted to have a left knee ulcer, which was treated with local wound care. Five months after transplant, the patient underwent work-up for chronic elevated serum creatinine, including an allograft biopsy which showed no evidence of rejection. However, lab studies revealed cytomegalovirus viremia, for which he was appropriately treated. Nine months after transplant he was readmitted with chills, fevers, and an elevated creatinine. The differential included allograft rejection, infection, and/or anatomic obstruction. He underwent a transplant kidney biopsy which showed only acute tubular injury consistent with tacrolimus toxicity. Blood cultures were positive for coagulase-positive staphylococcus, and he was treated for his bacteremia with a two-week course of appropriate antibiotics. Eleven months after transplant he was readmitted with fever, muscle weakness, joint pain, and myalgias. He was found to have a urinary tract infection and bacteremia with coagulase-positive staphylococcus and treated with a course of antibiotics. Rheumatology performed a diagnostic aspiration of his wrist due to concern for crystalline disease, but the aspirate was negative for crystals and the joint pain and myalgias were attributed to his bacteremia and lymphedema. He was discharged to a rehab facility. 

**Figure 2:**
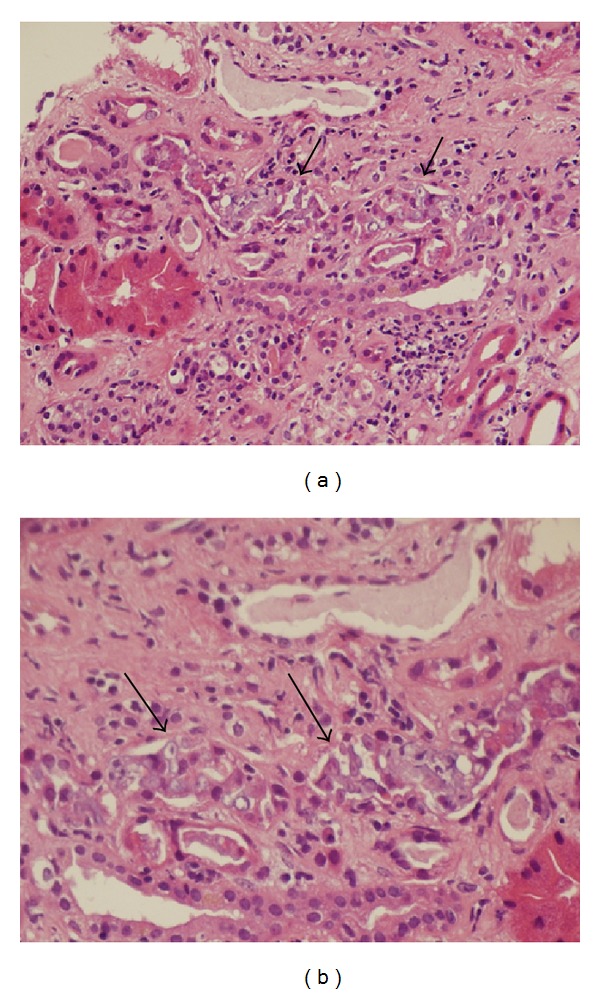
Case B renal allograft biopsy. Intratubular small fragments and hyphae of mucormycetes (by arrows) without extratubular invasion are noted by low magnification (a) and high magnification (b) on H&E staining sections.

Thirteen months after transplant he was seen as an outpatient, the left knee ulcer was noted to not be healing adequately and was biopsied in clinic. The biopsy specimen revealed cutaneous mucormycosis and he was admitted for treatment. He was started on amphotericin B and taken to the operating room for surgical debridement of his left knee lesion. He had an acute rise in his creatinine, which prompted a transplant kidney biopsy three days later, showing renal mucormycosis (Figure 2). 

 Due to the concern for disseminated mucormycosis, CT imaging of his head, neck, chest, abdomen, and pelvis were done which did not show any evidence mucormycosis in these sites. Due to his significant cardiac history, he first underwent a cardiac catheterization then subsequently underwent transplant nephrectomy. He was also found to have another cytomegalovirus infection which was treated with a course of valganciclovir. He was discharged to a long term care facility on postnephrectomy day 9 on a one month course of amphotericin B and then transitioned to oral posaconazole. He was seen for followup in transplant clinic one month later and was noted to have a healing left knee wound, being treated with a wound vac. He died at the rehabilitation facility from cardiac causes. 

## 3. Discussion

Renal mucormycosis is a rarely described and potentially lethal complication of kidney transplantation. Case A developed isolated renal mucormycosis and was presented early in the posttransplantation course, undergoing transplant nephrectomy eleven days after transplantation. Histopathology showed an invasion of mucormycosis in the transplanted kidney parenchyma and both donor and recipient ureter, but none in the graft artery or vein. This finding, combined with the fact that neither of the other two recipients of grafts from the same donor developed mucormycosis, suggests that this case of renal mucormycosis originated in the recipient rather than donor derived. However, another potential source of the mucormycosis could be from contaminated preservation fluid. Case B, in contrast, was presented late (more than a year after transplantation), initially manifested as cutaneous mucormycosis treated with debridement and amphotericin B, and found to have simultaneous renal mucormycosis, treated with transplant nephrectomy. In both cases, renal mucormycosis was an unanticipated finding, but one that was promptly acted upon with aggressive surgical debridement, including graft removal and appropriate antifungal agents. Prompt surgical intervention combined with appropriate antifungal therapy contributed to successful outcome in Case A. Case B, in contrast, had significant exposure to immunosuppression therapy as indicated by his persistent CMV infection and cutaneous mucormycosis. In addition, he had significant comorbidities known to increase the risk of Mucor infection including poorly managed insulin dependent diabetes mellitus and ischemic cardiomyopathy. 

*The Literature.* The first reported case of renal mucormycosis following kidney transplantation in the United States was in 2010. Alexander et al. described two patients who developed renal mucormycosis following kidney transplant from the same deceased donor [9]. Both patients required nephrectomies and treatment with amphotericin B, with one patient dying at posttransplant day 12 and the other patient surviving to discharge after an 84-day hospitalization. Histopathology of the explanted kidneys revealed vascular invasion with aseptate hyphae and relative sparing of the renal capsules, suggesting a vascular route of contamination. Genotypically indistinguishable strains of *Apophysomyces elegans* were recovered from both recipients but not established in the donor, suggesting either contamination of the organs during recovery or undiagnosed donor infection. 

In the world literature there are 14 additional international case reports of renal mucormycosis following renal transplantation, summarized in Table 1 [10–21]. 

 While it is necessary to review the world literature on renal mucormycosis following kidney transplantation, it is important to take into account differences between US and worldwide practices in immunosuppression, organ source, recipient surveillance, and graft management. Based on this sum of 18 reported cases worldwide (including our two cases), renal mucormycosis following kidney transplantation carries a staggeringly high 50% mortality rate. 

Overall, the majority of renal transplant patients who develop renal mucormycosis are male are diagnosed within the first couple months with the exception of our reported cases, and despite undergoing graft nephrectomy and systemic therapy with Amphotericin B they have a mortality of 50%. The risk factors include the use of immunosuppression drugs, diabetes, environmental factors, and the use of broad spectrum antimicrobial agents. Diagnosis of mucormycosis in transplant patients is extremely difficult and challenging because of rarity, lack of serologic tests, difficulty in isolation, and growth of mucormycetes from infected tissue, blood and body fluid, and often poor staining of mucormycetes with Grocott's methenamine silver stain (GMS) and Periodic Acid Schiff (PAS) due to possible mucoid features and sometimes mimicking acute cellular rejection in clinical presentation. 

Although extremely rare, renal mucormycosis is a severe and potentially lethal complication of renal transplantation. The keys to successful outcome are having a high index of suspicion for mucormycosis in the differential to enable early recognition, recognizing over immunosuppression as a risk factor for mucormycosis, and prompt, aggressive institution of surgical resection of infected tissue and appropriate pharmacological therapy in order to salvage patients with renal mucormycosis. A three-point strategy in the treatment of mucormycosis is essential and includes surgery, antifungal therapy, and management of risk factors.

## Figures and Tables

**Table 1:** Cases of renal mucormycosis following kidney transplantation.

Case report	R/D age	R/D sex	Donor type	Donor cause of death	Recipient disease	Time to transplant nephrectomy (PT)	Induction agent	Systemic therapy	Geographic location	Speciation	Outcome (PT)
S. Kuy et al.	36/43	M/F	SCD	Intracranial hemorrhage	Hemolytic uremic syndrome	11 days	Thymoglobulin, Solu Medrol	Amphotericin B	US	*Rhizomucor *	Survived
S. Kuy et al.	54/61	M/F	DCD ECD	Stroke	Diabetes mellitus, hypertension	13 months	Thymoglobulin, Solu Medrol	Amphotericin B	US	*Rhizomucor *	Survived
Alexander et al. [9]	61/17	M/F	DCD	MVC, drowning	—	9 days	Thymoglobulin, Solu Medrol	Amphotericin B	US	*Apophysomyces elegans *	Died (12 days)
Alexander et al. [9]	31/17	F/F	DCD	MVC, drowning	—	10 days	Thymoglobulin, Solu Medrol	Amphotericin B	US	*Apophysomyces elegans *	Survived
Mitwalli et al. [10]	18/—	F/—	LURD	—	Polycystic kidney disease	1 month	—	Amphotericin B	India	—	Survived
Nampoory et al. [11]	59/—	F/—	LURD	—	—	1 month	—	—	Kuwait	—	Died (3 months)
Stas et al. [12]	51/—	M/—	LURD	—	Henoch-Schonlein nephritis	2.5 months	—	—	India	*Absidia corymbifera *	Survived
Chkhotua et al. [13]	42/—	M/—	LURD	—	Glomerulonephritis	1.5 months	—	—	Egypt	*Mucorales *	Died (1.5 months)
Nalmas et al. [14]	62	M/—	LURD	—	—	3 weeks	—	—	Pakistan	*Mucor *species	Survived
Armaly et al. [15]	42/—	M/—	LURD	—	Poststreptococcal glomerulonephritis	1 month	—	—	Egypt	—	Died (1.5 months)
Armaly et al. [15]	52/—	F/—	LURD	—	Diabetes mellitus	1 month	—	—	Egypt	—	Survived
Godara et al. [16]	42/—	M/—	—	—	—	1 month		Amphotericin B	India	—	Died (1 month)
Sajiv et al. [17]	14/—	M/—	—	—	Congenital UPJ obstruction	3 months	Methylprednisolone, OKT3	Amphotericin B, Fluconazole	India	*Mucor *species	Died (few days)
Tayyebi et al. [18]	31/—	M/—	DCD	—	UPJ obstruction	2 months	—	—	Iran	*Mucor *species	Survived
Tayyebi et al. [18]	58/—	F/—	LURD	—	Polycystic kidney disease	9 months	—	—	Iran	*Mucor *species	Survived
Tomazic et al. [19]	56/28	M/M	LURD	—	—	2 months	Tacrolimus and Methylprednisolone	—	India	*Mucor *species	Died (2 months)
Minz et al. [20]	52/—	M/—	LURD	—	Chronic glomerulonephritis	8 days	Cyclosporin, azathioprine, prednisolone, Basiliximab	—	India	*Mucor *species	Died (8 days)
Ahmad [21]	49/—	M/M	LRRD	LRRD	Chronic glomerulonephritis	18 months	Cyclosporine, Prednisone, methylprednisolone, ATG	—	India	*Mucor *species	Died

R: recipient; D: donor; M: male, F: female; —: no data available; PT: after transplant; SCD: standard criteria donor; ECD: extended criteria donor, DCD: donor after cardiac death; LURD: living unrelated donor; MVC: motor vehicle crash; yrs: years old; UPJ: ureteropelvic junction; US: United States; ATG: antithymocyte globulin.
